# Supplementary material for: Photothermal Therapy with HER2-Targeted Silver Nanoparticles Leading to Cancer Remission
Source: Pharmaceutics. 2022 May 8;14(5):1013. doi: 10.3390/pharmaceutics14051013 (PMC9145338; doi:10.3390/pharmaceutics14051013)
Supplement: Supplementary file 1 [file pharmaceutics-14-01013-s001.zip › pharmaceutics-1697856-Supplementary.pdf]

pharmaceutics

MDPI

Supplementary Materials: Photothermal Therapy with HER2-Targeted Silver Nanoparticles Leading to Cancer Remission

Victoria O. Shipunova, Mariia M. Belova, Polina A. Kotelnikova, Olga N. Shilova, Aziz B. Mirkasymov, Natalia V. Danilova, Elena N. Komedchikova, Rachela Popovtzer, Sergey M. Deyev, Maxim P. Nikitin

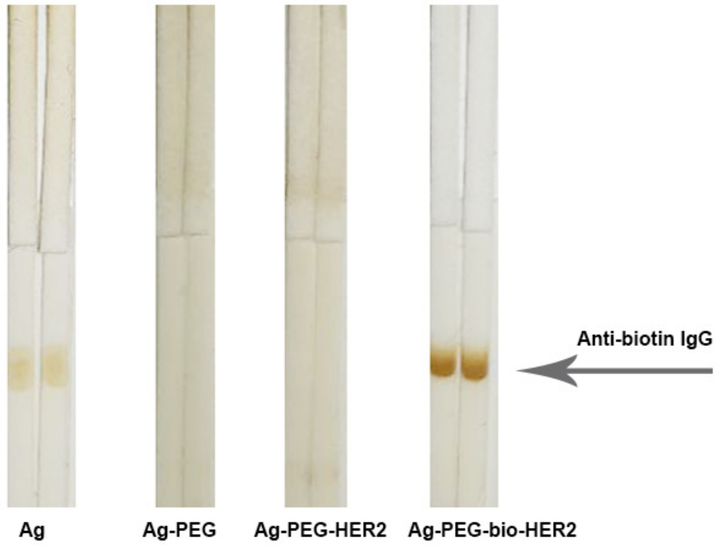

Figure S1. Immunochromatography assay on the conjugation efficiency. The anti-biotin IgG was applied to the nitrocellulose test strip. The strips were placed into the tube with: unmodified Ag NPs, PEG-modified AgNPs, AG-PEG-HER2 particles, and Ag-PEG-bio-HER2 particles (modified with biotin-labeled affibody).

Supporting Note 1
Methods
Affibody labeled with biotin was prepared as follows. 130 µL of affibody $Z_{HER2:342}$ in PBS at 1 g/L was mixed with 10 µL of biotin-NHS ester (Thermo Fisher, USA, EZ-Link NHS-Biotin) in DMSO at 8 g/L and incubated for 2 h at room temperature. The excess of unreacted molecules was removed with Zeba Spin Desalting Columns 7 kDa MWCO (Thermo Fisher, USA) according to the manufacturer's recommendations. The concentration of protein was determined with the BCA protein assay. The conjugation of biotin-labeled affibody with Ag-PEG particles was performed absolutely in the same way as for pristine affibody.
For immunochromatograpy test, the 2 mm test strip (MDI, # LKDFXXX060X260X 90CNPH-N-SS40-L2-P25) was applied with 1 µL of anti-biotin IgG (IgG Fraction Monoclonal Mouse Anti-Biotin, Jackson Immunoresearch) at 1.2 g/L and air-dried. Next, the strip was placed into the tube with 40 µL Tris, 1% BSA, 0.05% Tween-20 and 2 µg of test particles. The particles were allowed to migrate until the all liquid was absorbed on absorbent pad. The strips were imaged with a smartphone camera.

Results
To validate the presence of protein – affibody $Z_{HER2:342}$ on the nanoparticle surface, we performed a lateral flow assay with immunochromatograpy strips coated with anti-biotin IgG and particles conjugated to the biotinylated affibody, namely, Ag-PEG-bio-HER2.
The data presented in Fig. S1 demonstrate that uncoated Ag NPs possess slight non-spesific binding to anti-biotin IgG. However, this non-spesific binding is successfully shielded by modification of nanoparticle surface with PEG (ag-PEG). The affibody-conjugated Ag-PEG-HER2 particles do not exhibit any binding to anti-biotin

IgG. And only the biotin-affibody conjugated Ag-PEG-bio-HER2 particles interact specifically with the anti-biotin IgG thus forming a strong absorbing line on the test strip thus confirming the efficiency of conjugation.

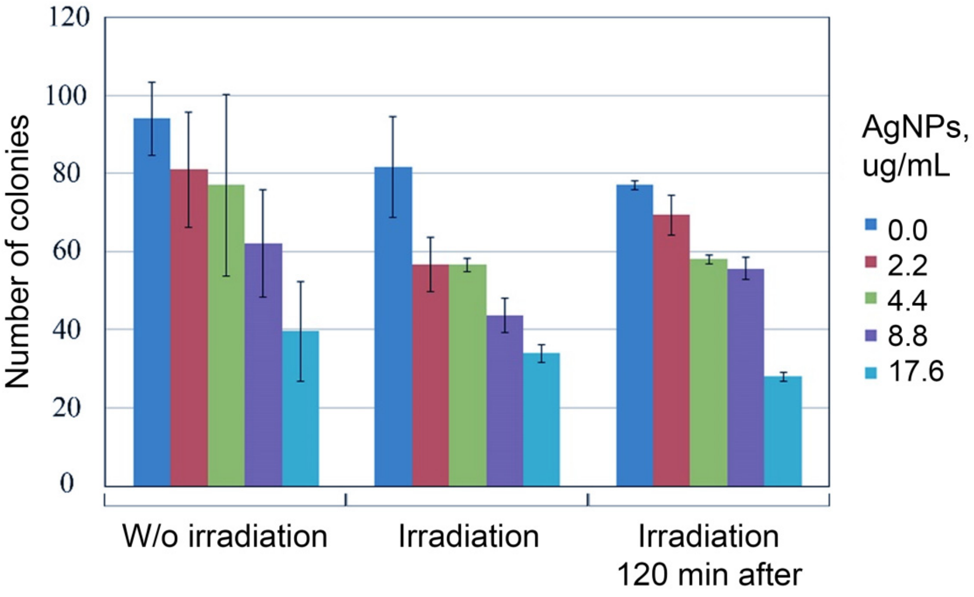

Figure S2. Clonogenic assay. The number of SKOV3-1ip cell colonies depending on the Ag-PEG-HER2 concentration and irradiation options with an LED matrix at a power of 95 mW/cm^2.

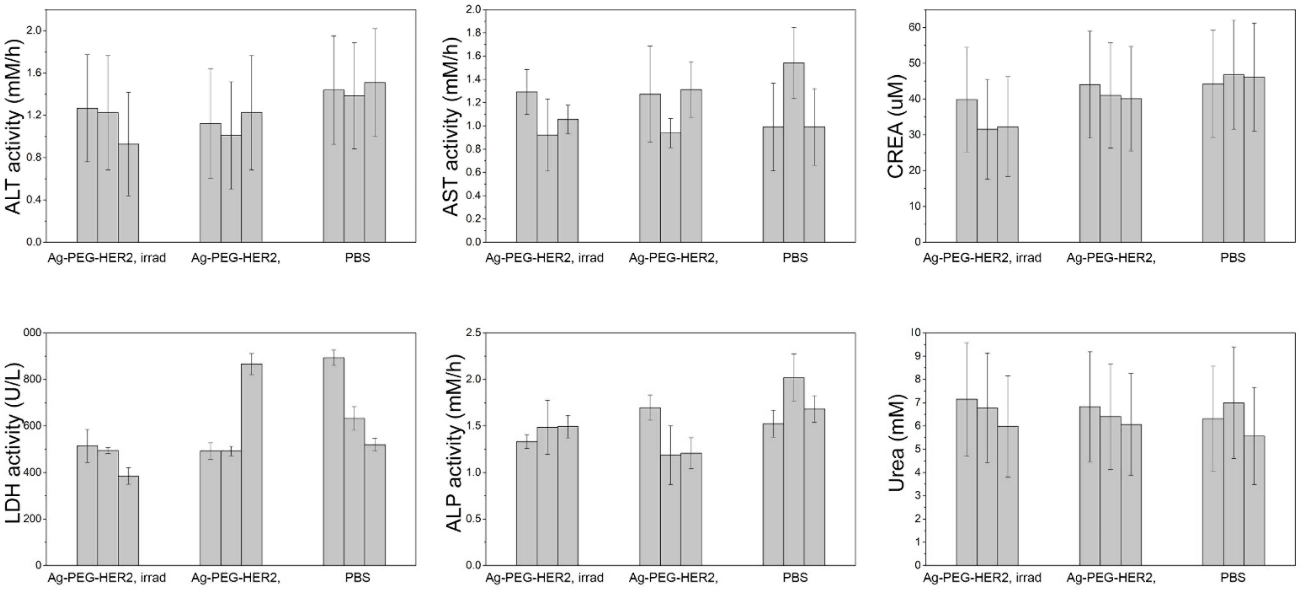

Figure S3. Safety aspects of Ag-based PTT. The levels of various blood biochemical parameters after the Ag-PEG-HER2 treatment in three experimental groups that received the injections of i) group 1 – Ag-PEG-HER2 nanoparticles followed with light irradiation, ii) group 2 – Ag-PEG-HER2 nanoparticles, iii) group 3 – PBS (control group). Liver function was determined by serum activity of alanine aminotransferase (ALT) and aspartate aminotransferase (AST). Nephrotoxicity was assessed by creatinine (CREA) and urea levels. Cardiac damage was assessed by lactate dehydrogenase (LDH) activity. Alkaline phosphatase (ALP) activity is an indicator of hepatobiliary or bone damage.

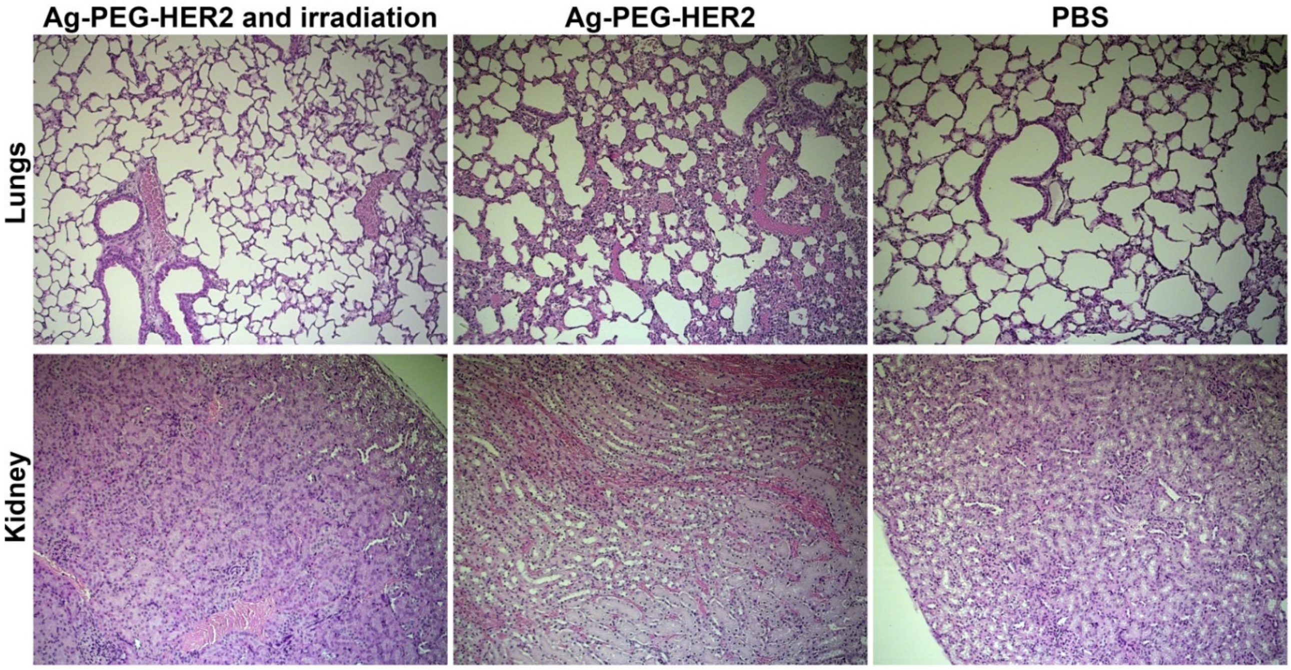

Figure S4. Histology study. Hematoxylin and eosin staining of lungs and kidneys of mice from three experimental groups that received the injections of i) group 1 – Ag-PEG-HER2 nanoparticles followed with light irradiation, ii) group 2 – Ag-PEG-HER2 nanoparticles, iii) group 3 – PBS (control group). Magnification, 100x.
